# Life-history features and oceanography drive phylogeographic patterns of the chiton *Acanthochitona* cf.* rubrolineata* (Lischke, 1873) in the northwestern Pacific

**DOI:** 10.7717/peerj.8794

**Published:** 2020-04-08

**Authors:** Gang Ni, Taeho Kim, Youngheon Shin, Jina Park, Yucheol Lee, Hyun-Jong Kil, Joong-Ki Park

**Affiliations:** 1 Division of EcoScience, Ewha Womans University, Seoul, Republic of Korea; 2 Department of Biological Sciences, Sungkyunkwan University, Suwon, South Korea; 3 Animal Resources Division, National Institute of Biological Resources, Incheon, Republic of Korea

**Keywords:** Marine phylogeography, *Acanthochitona*, Polyplacophora, Limited gene flow, Northwestern Pacific

## Abstract

Chitons are a group of marine mollusks (class Polyplacophora) characterized by having eight articulating shell plates on their dorsal body surface. They represent suitable materials for studying the spatiotemporal processes that underlie population differentiation and speciation in ocean environments. Here we performed population genetic analyses on the northwestern Pacific chiton *Acanthochitona* cf. *rubrolineata* (Lischke, 1873) using two mitochondrial gene fragments (COI and 16S) from 180 individuals sampled from 11 populations among the coastal waters of Korea, Japan, and China. The phylogenetic network uncovered a reticulated relationship with several sub-haplogroups for all *A.* cf. * rubrolineata* haplotypes. SAMOVA analyses suggested the best grouping occurred at three groups (Φ_CT_ = 0.151, *P* < 0.0001), which geographically corresponds to hydrographic discontinuity among the coastal regions of Korea, Japan, and China. The assumed limited dispersal ability of * A.* cf. * rubrolineata*, coupled with northeasterly flowing, trifurcate warm currents, might have contributed to the genetic differentiation among the three groups. Meanwhile, a high level of within-group genetic homogeneity was detected, indicating extensive coastal currents might facilitate gene flow among the populations within each group. Bayesian skyline plots demonstrated significant population expansion after the Last Glacial Period (110-25 thousand years ago) for all studied populations except the Japan group. Together these results suggest that the present-day phylogeographic patterns of *A.* cf. *rubrolineata* are strongly affected by the interplay of historical and/or contemporary oceanography and species-specific life-history features.

## Introduction

The northwestern Pacific (NWP) is a vast region comprising the coastlines of China, Korea, Japan, and Russia. It features a unique tectonic and hydrological setting and provides an ideal system to test some interesting biogeographic questions that underlie marine species’ evolution (Liu et al., 2007; Ni et al., 2014). During recent years, many phylogeographic studies on diverse species have revealed the impacts of different environmental factors in the NWP region (e.g., Guo et al., 2015; Han et al., 2015; Li et al., 2017; Ni et al., 2012; Zhao et al., 2018). However, most of these studies have focused on cryptic lineages promoted by separation of marginal seas during the Pleistocene (e.g., Liu et al., 2011; Qiu et al., 2016; Wang et al., 2017; Xu et al., 2009), leaving the effects of some other biotic and/or abiotic factors largely unexplored (Ni et al., 2015b). Oceanographic features, habitat discontinuity, and species-specific life history can also contribute to shaping population genetic structure and patterns in various marine species (Dong et al., 2012; Ni et al., 2017).

**Figure 1:**
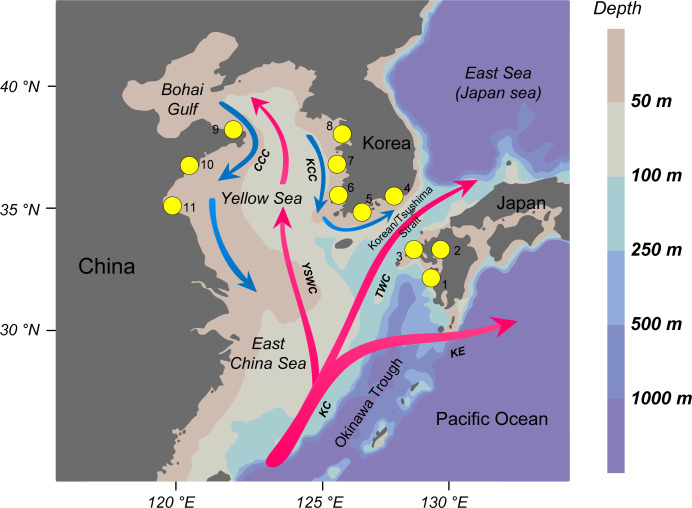
The northwestern Pacific map. Map showing the sampling sites of *Acanthochitona* cf. *rubrolineata* and the ocean currents operating in this portion of the northwestern Pacific. The warm currents are shown in pink (KC, Kuroshio Current; TWC, Tsushima Warm Current; YSWC, Yellow Sea Warm Current; KE, Kuroshio Extension), while the coastal currents are shown in blue (CCC, China Coastal Current; KCC, Korea Coastal Current).

The Yellow Sea (YS), including the Bohai Gulf, is a marginal sea of the NWP located between the west coast of Korea and the east coast of China (Fig. 1). With a total area of 458,000 km^2^, it is characterized by an extensive continental shelf with an average depth of 55 m (Park & Yi, 1995; Wang, 1999). Past climate changes during the Pleistocene might have dramatically impacted the environments and tectonic configurations of the YS. When glaciers advanced during glacial periods, the sea level was about 120 m lower than the present-day, resulting in the complete exposure of the YS shelf (Wang, 1999; Wang & Wang, 1980). Marine populations inhabiting these coastal regions, especially those of macrobenthic species, were exterminated or forced to migrate southeastward and survived in the assumed refugium of the Okinawa Trough (Liu et al., 2011; Ni et al., 2015a). When the sea level rose during interglacial periods, surviving refugial populations repopulated the newly-formed coasts of the YS (Han et al., 2015; He et al., 2010; Liu et al., 2007). These populations were presumably genetically homogeneous because they would have originated from a panmictic ancestral population in the trough after the last glacial maximum (Liu et al., 2011).

Present-day oceanographic features also shape phylogeographic patterns of marine species (He et al., 2015; Li et al., 2017). Two ocean current systems, including a coastal current system and a warm current system, are operating in the YS. Several main coastal currents flow along the coastlines: the Korea Coastal Current flows from north to south along the western coast of the Korean peninsula; and the China Coast Current begins in the Bohai Sea and flows southward along the Chinese coast (Guan, 1994; Su & Yuan, 2005). Additionally, there are several branches of the Kuroshio Current in this region, including the Yellow Sea Warm Current flowing between northern China and the Korea peninsula, and the Tsushima Warm Current flowing through the Korean/Tsushima Strait, separating the Korean peninsula and the Japanese islands (Isobe, 1999; Su & Yuan, 2005).

*Acanthochitona* is a genus of the family Acanthochitonidae (Polyplacophora) and is distributed worldwide. It is one of the most taxonomically complicated molluskan groups due to its extremely high morphological variability (Bonfitto et al., 2011; Taki, 1938). Some *Acanthochitona* species are abundant in coastal areas of the NWP (Hong & Richard, 1990; Saito, 2017), providing good materials for understanding the spatiotemporal processes that underlie marine population differentiation and speciation. In this study, we focus on phylogeographic patterns of a chiton species *A.* cf. *rubrolineata* (Lischke, 1873) in the NWP. Although our identifications correspond to the conventional use of *A.* cf. *rubrolineata* for this species, Eernisse, Draeger & Pilgrim (2018) found that it is part of a species complex of at least six species of this genus and associated the name with a different species than we do. In their work, *A*. sp. B *sensu* Eernisse, Draeger & Pilgrim (2018) corresponds to our *A*. cf. *rubrolineata*. They associated *A.* cf. *rubrolineata* with a different species based on comparisons with Taki’s (1938) description and also based on the sequences of Russian specimens identified as that species by B. Sirenko (DJ Eernisse, 2019, pers. comm.). The assignment here is primarily based on the fact that we had the opportunity to sequence the first eight specimens sampled from the vicinity of the “Nagasaki Japan” type locality for *A. rubrolineata*, but we agree with them that it is still necessary to compare Lischke’s (1873) type material to both species, and also further sample the Nagasaki region to determine whether both species might be present in the vicinity of the type locality.

According to existing literature and our field sampling, *A.* cf. *rubrolineata* has a wide distribution in the NWP from Kyushu, Japan, to the southern and western coasts of Korea, to the northern coast of east China (Saito, 2017). The chitons are often found on hard surfaces like rocks (or in rock crevices) in intertidal zones, usually forming an intertidal community with other macrobenthic species such as *Mytilus edulis* or *Corallina pilulifera* (Hong & Richard, 1990). There is no direct information about the larval development of *A.* cf. *rubrolineata*, but evidence from a wide range of chiton species suggests an overall short planktonic time of < one week (Pearse, 1979). *A.* cf. *rubrolineata* is therefore assumed to have low dispersal capacity that may contribute to significant population structure among populations. However, how these life-history characteristics have acted in concert with ocean current systems to shape phylogeographic patterns in *A.* cf. *rubrolineata* still warrants a detailed genetic appraisal.

In this study, we determined the sequences of two mitochondrial genes [cytochrome oxidase subunit I (COI) and 16S ribosomal DNA (16S)] for 180 NWP individuals of *A.* cf. *rubrolineata*, including 102 specimens sampled from the southern coastal areas of the Korean Peninsula, 18 specimens from populations in the Nagasaki Prefecture, Kyushu Island, Japan (in the vicinity of the “Nagasaki” type locality for *A. rubrolineata*), and 60 specimens from northeastern China, in order to shed light on the phylogeographic patterns of this species. We aimed to evaluate (1) the impact of a known history of sea-level changes during the Pleistocene on the present-day phylogeographic pattern of this chiton species, (2) population structure for a chiton species, which belongs to a marine molluskan taxon characterized by lecithotrophic development and limited planktonic larval duration; and (3) gene flow among the three regions and the influence of ocean currents on contemporary population structure.

## Material and Methods

### Sampling, DNA extraction, PCR and sequencing

A total of 180 *A.* cf. *rubrolineata* individuals were sampled from 11 localities in Korea, Japan, and China, and about 20 specimens per population except for the Japanese populations were sequenced for mitochondrial COI and 16S (Fig. 1 and Table 1). Collected samples were fixed and kept in 95% ethanol until DNA extraction. Genomic DNA was extracted using the E.Z.N.A. Mollusc DNA Kit (OMEGA Bio-tek, Norcross, GA, USA) following the manufacturer’s protocol. Two fragments of mitochondrial COI and 16S were amplified using the universal primer sets of LCO1490 (5′-GGT CAA CAA ATC ATA AAG ATA TTG G-3′) and HCO2198 (5′-TAA ACT TCA GGG TGA CCA AAA AAT CA-3′) (Folmer et al., 1994), and 16Sar (5′-CGC CTG TTT ATC AAA AAC AT-3′) and 16Sbr (5′-CCG GTC TGA ACT CAG ATC ACG T-3′) (16S; Palumbi, 1996). The polymerase chain reaction (PCR) was performed in a 50 µl volume containing 1.5 µl of template DNA, 5 µl of 10x Ex Taq buffer (Takara, Shiga, Japan), 4 µl of 0.2 mM dNTP mixture, 1 µl of each primer (10 pmole), and 0.25 µl Ex Taq Polymerase. The amplification conditions were initial denaturation at 95 °C for 1 min, followed by 40 cycles of denaturation at 94 °C for 30 s, annealing at 46 °C for 30 s for COI and 52 °C for 30 s for 16S, extension at 72 °C for 30 s, and a final extension at 72 °C for 10 min. The PCR product was purified with a QIAquick Gel Extraction Kit (Qiagen, Valencia, CA, USA) and then sequenced using an ABI PRISM 3730xl DNA analyzer (Applied Biosystems, Foster City, CA, USA) in both directions.

**Table 1:** Sampling information and diversity indices for each population of *Acanthochitona* cf. *rubrolineata*.

Locality (Abbreviation)	Latitude, longitude	*N*	*Nh*	*Hd*	*Nd*	*Pd*
1. Nomomachi, Kyushu, Japan (NO)	32°35′N, 129°45′E	3	3	1	0.0046	5.333
2. Nabegushimen, Kyushu, Japan (NA)	33°24′N, 129°47′E	8	7	0.964	0.0094	10.964
3. Kinaisemen, Kyushu, Japan (KI)	33°22′N, 129°51′E	7	7	1	0.0098	11.333
4. Yeosu, Jeollanam-do, Korea (YE)	34°47′N, 127°45′E	20	19	0.995	0.0079	9.179
5. Wando, Jeollanam-do, Korea (WA)	34°19′N, 126°44′E	22	18	0.978	0.0078	9.009
6. Buan, Jeollabuk-do, Korea (BU)	35°37′N, 126°27′E	20	17	0.984	0.0085	9.837
7. Taean, Chungcheongnam-do, Korea (TA)	36°23′N, 126°25′E	20	13	0.905	0.0081	9.453
8. Ansan, Gyeonggi-do, Korea (AN)	37°11′N, 126°32′E	20	11	0.805	0.0064	7.437
9. Weihai, Shandong, China (WE)	37°32′N, 122°09′E	20	11	0.895	0.0087	10.089
10. Qingdao, Shandong, China (QI)	36°02′N, 120°21′E	20	9	0.753	0.0050	5.821
11. Lianyungang, Jiangsu, China (LI)	34°45′N, 119°29′E	20	10	0.758	0.0068	7.937

**Notes.**

*N* number of individuals *Nh* number of haplotypes *Hd* haplotype diversity *Nd* nucleotide diversity *Pd* mean number of pairwise differences

### Sequence analyses

Sequences were assembled and edited in Geneious (Kearse et al., 2012) and then deposited in GenBank (accession numbers: 16S: MN205571–MN205750; COI: MN205751–MN205930). We merged the two mitochondrial segments for each individual using the software FasParser (Sun, 2017), and performed a partition homogeneity test in PAUP* 4.0 b10 (Swofford, 2002) to check the congruence between COI and 16S sequences with the heuristic search option (number of replicates = 100). The result (*P*-value = 0.680) suggested there was no incongruence between the two segments, and therefore the concatenated sequences were used in subsequent analyses. Haplotypes were defined using DnaSP v5 (Librado & Rozas, 2009), and their relationships were inferred using the TCS network in Popart v1.7 (Leigh & Bryant, 2015). The software jModelTest 2 (Darriba et al., 2012) was used to determine the best-fit model for sequence evolution. HKY+I was selected as the best model based on Bayesian Information Criterion and used in the following sets. Genetic diversity indices, including haplotype diversity (*Hd*), nucleotide diversity (*Nd*), and mean number of pairwise differences (*Pd*) were calculated for each of the 11 populations in ARLEQUIN v3.5 (Excoffier & Lischer, 2010).

For population structure analyses, population clusters were first estimated for 10 populations (excluding the Nomomachi population in Japan, because only three individuals were analyzed; see Table 1) based on *F*_CT_ values using SAMOVA 2.0 (Dupanloup, Schneider & Excoffier, 2002). The test was performed with K-values of 2-7, and the value for which the *F*_CT_ was highest was chosen as the best grouping. Based on the cluster identified from the SAMOVA, analysis of molecular variance (AMOVA) was then conducted with 10,000 permutations in ARLEQUIN v3.5 to estimate the partitioning of genetic variation. Since the HKY model was not available in ARLEQUIN, the most similar model, that of Tamura-Nei (Tamura & Nei, 1993) was used to estimate population structure. Under the same model, pairwise population comparisons (*F*_ST_ values) were calculated with 10,000 permutations in ARLEQUIN v3.5.

We used the coalescent-based program MIGRATE-N v. 3.6 (Beerli & Felsenstein, 1999; Beerli & Palczewski, 2010) to estimate mutation-scaled migration rate (*M*) among the groups defined in SAMOVA and effective population size (Θ) of each group based on maximum-likelihood estimates. Several short runs were performed to check for convergence of chains, and appropriate upper bounds were set for each parameter after the test runs. Three long chains were run with 2,000,000 steps and sampled every 100 steps, and the first 20,000 steps were discarded as burn-in. A static heating scheme was set at four temperatures of 1, 1.5, 3, and 100,000. Five replicates were run to check the consistency of the estimates, and stationarity of the Markov chain Monte Carlo (MCMC) was assessed by examining the effective sample size (ESS). We transformed the migration rate to the number of migrants per generation (*Nm*) using the formula *Nm* = Θ*M/x (where x is the inheritance parameter, and here *x* = 1 for mitochondrial gene) (Beerli & Palczewski, 2010).

Neutrality tests were applied to each group defined in SAMOVA and the entire population (including all the sampled individuals) to infer historical demography. Tajima’s *D* (Tajima, 1989) and Fu’s *Fs* (Fu, 1997) tests were conducted with 10,000 replications in ARLEQUIN v3.5. In addition, a mismatch distribution analysis was performed using DnaSP v5 to test the signal of historical expansion. Bayesian skyline plot (BSP) analysis was performed for the COI data set using BEAST v2.4.8 (Bouckaert et al., 2014) to infer historical demography of effective population size for the entire population and individual groups as defined in SAMOVA. We selected the HKY model with four rate categories of heterogeneity as the site evolution model. For the clock model, we used a log-normal relaxed clock. There is no reliable molecular clock yet for mitochondrial COI sequences of this chiton species. However, a molecular clock for the mtDNA COI of other two *Acanthochitona* species was estimated, ranging from 1.0 to 2.0% per million years (myr) based on the minimum splitting time between Caribbean and Eastern Pacific presumed geminate species pairs of the Isthmus of Panama (Ríos et al., 2014). Here we applied the lower bound of 1.0%/myr for the COI sequences and a generation time of two years (Avila-Poveda & Abadia-Chanona, 2013; Cherns, 1999) to convert the parameters to real demographic times. As the priors, the Coalescent Bayesian Skyline model was chosen while setting the free standard deviation (ucldStdev) parameter to Exponential. The analysis was performed with an MCMC chain length of 20 million iterations for the dataset including all individuals, and 10 million for each group, sampling every 1,000 generation to estimate the effective population size change. Tracer 1.6 was used to check and visualize the results (available from http://beast.bio.ed.ac.uk/Tracer). The 16S data set was not used here because it is uninformative and there is no reported molecular clock yet for this species or any other *Acanthochitona* species.

## Results

**Figure 2:**
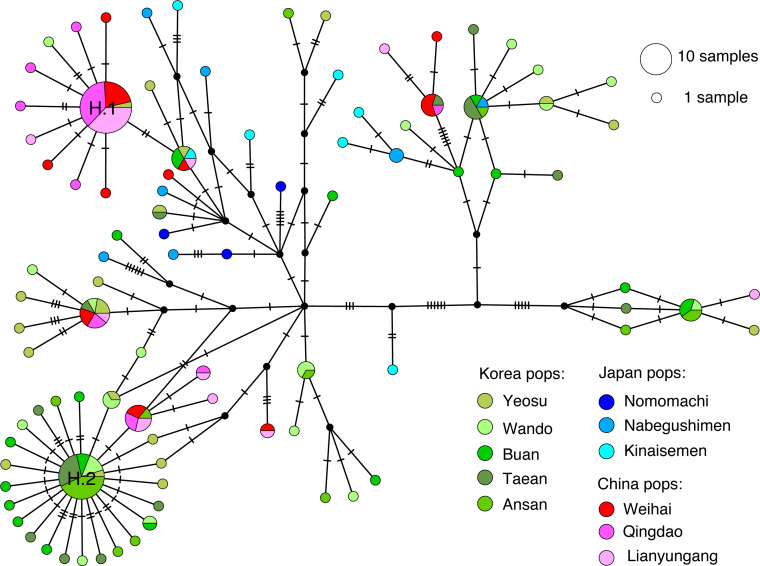
Haplotype network. TCS network showing the relationship among all the haplotypes and the distribution of haplotypes in each population. The circle size is proportional to the observed haplotype frequency. The two most abundant haplotypes are labeled with H.1 and H.2, respectively.

COI and 16S gene data were obtained from the 180 examined individuals, and the final alignment of the concentrated sequences per individual was 1,161 sites long. A total of 114 variable sites were identified, yielding 92 haplotypes. The number of haplotypes in each population ranged from three in Nomomachi to 19 in Yeosu. The overall haplotype diversity was 0.958, ranging from 0.753 (in Qingdao, China) to 1.000 (in Nomomachi and Kinaisemen, Japan). Nucleotide diversity ranged from 0.0046 (in Nomomachi) to 0.0098 (in Kinaisemen), and the mean number of pairwise differences ranged from 5.333 to 11.333 (Table 1). The TCS network for all the haplotypes displayed a reticulated topology with several sub-haplogroups (Fig. 2). Some haplotypes were clustered within a specific region: for example, haplotype 1 dominated Chinese populations while haplotype 2 was widely distributed in all Korean populations.

In the SAMOVA analysis, the highest *F*_CT_ value occurred at *K* = 3 with a grouping arrangement of (NA, KI; Japan), (YE, WA, BU, TA, AN; Korea), and (WE, QI, LI; China), which geographically aligns with the three studied countries. Under this scenario, AMOVA analysis showed that both variation among groups (Φ_CT_ = 0.151) and variation within populations (Φ_ST_ = 0.154) were statistically significant (both *P*-values < 0.05), while variation among populations within groups was not significant (*P*-value = 0.5537). Variation within populations accounted for the largest percentage (84.91%) of the total variation, followed by variation among groups (15.38%), whereas a slightly negative variance value (−0.29%) was estimated for among populations within groups (Table 2). The pairwise population comparison showed that 21 out of 55 *F*_ST_ values were statistically significant (*P* < 0.05), and all of these were observed between populations of different groups (Table 3). In contrast, the *F*_ST_ values between populations of the same group were much lower and/or in some cases, not statistically significant (Table 2).

**Table 2:** AMOVA analysis. Analysis of molecular variance (AMOVA) for the three regional groups defined from SAMOVA analysis. Significant *P-* values are indicated in bold.

Grouping	Source of variation	*df*	Sum of squares	Variance components	Percentage of variation	Statistics	*P*-value
(NA, KI); (YE, WA, BU, TA, AN); (WE, QI, LI)	Among groups	2	86.499	0.80758	15.38	Φ_CT_ = 0.151	**<0.0001**
	Among populations within groups	7	29.243	−0.01522	−0.29	Φ_SC_ = 0.003	0.5537
	Within populations	167	744.487	4.45801	84.91	Φ_ST_ = 0.154	**0.0004**

**Table 3:** Pairwise comparisons. Pairwise *F*_ST_ values between 11 populations. The values in bold are statistically significant (*P*-value < 0.05).

Locality	1. NO	2. NA	3. KI	4. YE	5. WA	6. BU	7. TA	8. AN	9. WE	10. QI
1. NO										
2. NA	0.075									
3. KI	0.035	−0.094								
4. YE	0.051	0.073	0.043							
5. WA	0.132	0.044	0.025	0.001						
6. BU	0.124	0.047	0.032	0.013	−0.022					
7. TA	**0.180**	0.058	0.051	0.041	−0.018	−0.021				
8. AN	0.202	**0.163**	**0.130**	0.038	0.010	−0.015	0.010			
9. WE	0.054	0.072	0.042	0.057	**0.101**	**0.106**	**0.131**	**0.167**		
10. QI	0.207	**0.260**	**0.222**	**0.169**	**0.248**	**0.249**	**0.295**	**0.306**	0.029	
11. LI	0.094	**0.153**	**0.116**	**0.087**	**0.151**	**0.145**	**0.193**	**0.193**	−0.010	−0.027

**Figure 3:**
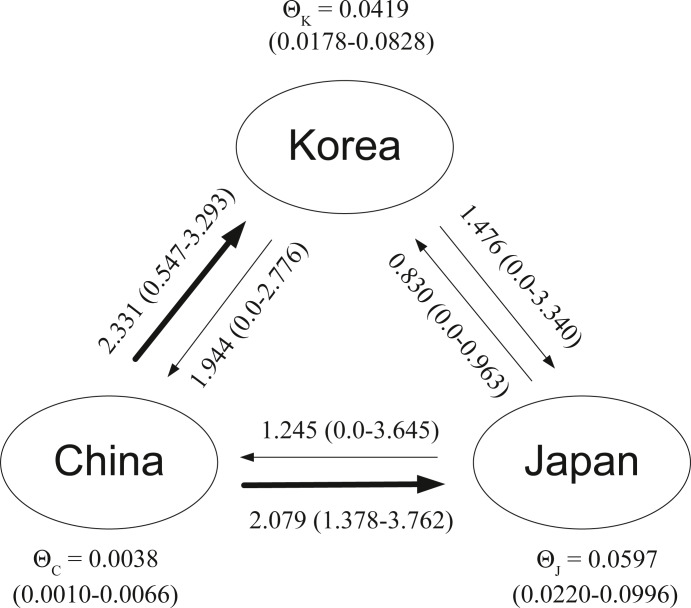
MIGRATE-N result. Results from MIGRATE-N for the three groups of *Acanthochitona* cf. *rubrolineata*, showing parameters of mutation-scaled effective population size and gene flow with the 95% confidence interval in brackets. Directions with significant gene flow are indicated with thicker arrows.

For the gene flow analyses in MIGRATE-N, the effective MCMC sample size of each parameter was >2000, indicating stationarity of all the chains. The results revealed asymmetric gene flow among the three groups (Fig. 3): significant migration was observed from the China group into the Korea group [*Nm*_(C→K)_: 2.331 with 95% confidence interval (CI : 0.547–3.293) and Japan group [*Nm*_(C→J)_: 2.079 (CI : 1.378–3.762)]. For the other four directions, the estimated values were relatively smaller and had a 95% CI that overlapped with zero, a signal that suggests impeded gene flow between pairwise groups. Effective population size with a 95% CI for each group revealed the smallest Θ in the China group [Θ_C_ = 0.0038 (0.0010–0.0066)], with comparable values for the Korea [Θ_K_ = 0.0419 (0.0178–0.0828)] and Japan [Θ_J_ = 0.0597 (0.0220–0.0996)] groups.

**Figure 4:**
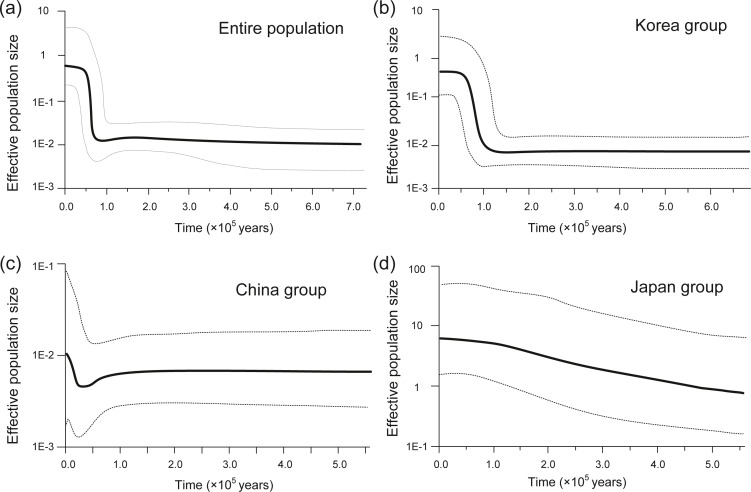
Bayesian skyline plots of historical demography of the entire population (A), Korea Group (B), China Group (C), and Japan Group (D), respectively. The mean value (solid line) with 95% highest posterior density (dotted line) is shown.

Different signals of population size change were observed in various demographic analyses for the entire population and the three regional groups. In the Tajima’s *D* test, all the analysis showed negative values, but *P* values were not significant in the China or Japan groups (Table 4). All the Fu’s *F*_*S*_ tests also showed negative and significant values except the China group. Mismatch distribution analyses revealed that all the groups showed a multimodal distribution pattern, indicating that the population size of the entire population and each regional group was at a demographic equilibrium (Fig. S1). In contrast, however, the BSP results revealed significant signals of sudden population size expansion (Fig. 4). The entire population and the Korea group showed a similar demographic trend: their population size dramatically increased in the recent past (60–70 kya (the entire population) and 100 kya (Korea group)), after a long period of population stasis (Figs. 4A, 4B). The China group showed a sudden population expansion in more recent past, beginning its expansion about 25 kya (Fig. 4C). The trajectory of the effective population size of the Japan group showed that its population size has been gradually increasing since 550 kya and differed from the trajectories of the other two regional groups (Korea and China) (Fig. 4D).

**Table 4:** Neutrality tests for the three groups. Results from neutrality tests (Tajima’s *D* and Fu’s *Fs*) for the entire population and each of the three regional groups (*P*-value <0.05 is indicated in bold.

Group	Tajima’s *D*			Fu’s *Fs*	
	*D*	*P*		*Fs*	*P*
1. All	−1.611	** 0.017**		−24.279	** <0.001**
2. Korea	−1.466	**0.040**		−24.358	**<0.001**
3. China	−0.712	0.260		−1.052	0.397
4. Japan	−0.759	0.229		−7.401	**0.005**

## Discussion

### Significant population structure among three regional groups

A most striking pattern in *A.* cf. *rubrolineata* is the existence of significant population structure among the populations suggested by the various analyses. The best grouping method in SAMOVA divided all study populations into three groups, which geographically correspond to the coastal regions of Korea, China, and Japan (Table 2). Pairwise *F*_ST_ estimates also showed that all significant values came from the comparison of populations between different groups (Table 3). Estimations in MIGRATE-N revealed asymmetric gene flow among the three groups: among the estimations of six possible directions of gene flow, only the directions from China to the other two regional groups were significant (Fig. 3). These results indicate that genetic connectivity (i.e., gene flow) is limited or at least partially impeded among populations of the three regions, contributing to significant population structure among them. A comprehensive review of the correlation between pelagic larval duration and dispersal distance of marine species has also shown that species with a larval stage of less than a week usually disperse a short span of 1–10 km (Shanks, 2009). Larval behavior (especially larval stage duration time) has been shown to play a critical role in determining dispersal distance for many marine species (Cowen & Sponaugle, 2009; Hellberg et al., 2002; Pineda, Hare & Sponaugle, 2007). Thus, it is not surprising to observe here significant population structure in *A.* cf. *rubrolineata* populations, considering its limited dispersal capacity (inferred from the life-history traits of many chiton species). Fertilized chiton eggs are known to hatch out after some period that mostly depends on temperature into trochophore larvae (ciliated, free-swimming larvae) and then become increasingly benthic and soon metamorphose into a juvenile form within a few days to about 12 days after fertilization depending on chiton species (Pearse, 1979). From the developmental study of a congeneric species (*A. crinitus*) that undergoes metamorphosis within 3-4 days after fertilization (Fritsch et al., 2015), *A.* cf. *rubrolineata* is assumed to have a short planktonic larval duration (albeit variable depending on temperature), likely preventing long-distance dispersal (i.e., gene flow) of *A.* cf. *rubrolineata* populations across our three studied regions. A short larval stage would likely affect population structure among the three regions, especially considering their geographic separation and habitat discontinuity, and the general lack of stepping stone habitats between them.

### Contribution of the warm current system to population structure among the regional groups

Although the magnitude of gene flow during the planktonic larval stage seems limited for chitons (Ríos et al., 2014; Todt et al., 2008), they can still be transported via rafting on floating macroalgae or terrestrial vegetation like trees (Eernisse, Draeger & Pilgrim, 2018). A long-distance dispersal (up to 600 km) by kelp rafting has been reported for two species of subantarctic chitons that dispersed their propagules from subantarctic islands to New Zealand (Fraser, Nikula & Waters, 2010). However, dispersal by rafting in *A.* cf. *rubrolineata* may be limited by the warm current system in the YS. The trifurcated branches of the Kuroshio Current (including the Yellow Sea Warm Current, the Tsushima Warm Current, and Kuroshio Extension) flow northeastward into their downstream pathways that separate the three regional groups. This oceanographic surface flow may act as a physical barrier that, albeit not completely, limits the opportunity for gene flow via larval dispersal and/or occasional rafting of the individuals across the three regions. Note that, as inferred from the MIGRATE-N results, this barrier effect may be weak for the directions from China to both Korea and Japan. Similar population structuring resulting from hydrographic patterns has also been reported in other marine species in the NWP (Ni et al., 2017) and other ocean systems, including the snakeskin chiton *Sypharochiton pelliserpentis* in New Zealand (Veale & Lavery, 2011) and coral reef fishes in the Caribbean (Taylor & Hellberg, 2006).

### Genetic homogeneity within the regional groups

In contrast to inter-regional genetic structuring proposed to be driven by the three branches of the Kuroshio Current, substantial genetic homogeneity was revealed within each regional group, with no significant population structure detected (Table 3). Genetic connectivity in each group is assumed to be facilitated by the present-day surface currents along the coastline of each region (Fig. 1). These coastal currents may play a significant role in maintaining genetic connectivity that contrasts with the warm currents: they are expected to enhance gene flow by transporting planktonic larvae and/or rafting adults among local populations, reducing genetic differentiation among the within-group populations. Previous studies have revealed a close correlation between the coastal current system and population homogeneity in many organisms in the YS (e.g., Li et al., 2010; Ni et al., 2015b; Xue et al., 2014). Relatively short distances between populations and habitat continuity within each region are also likely potential factors underlying their genetic homogeneity.

### Demographic history and the impact of Last Glacial Period

In estimating the historical population demography of *A.* cf. *rubrolineata*, results both from the neutrality test and BSP analysis supported a sudden historical population expansion. By reconstructing effective population size change in BSP, the estimated timing of population expansion for the entire population and Korea group were calculated to be 60–70 kya (the entire population) and 100 kya (Korea group) (Figs. 4A, 4B), after and/or approximately corresponding to the onset of Last Glacial Period (LGP, 110 to 15 kya), the most recent glacial period within the Quaternary Ice Age (Severinghaus & Brook, 1999). Abrupt climate change and subsequent sea level fluctuation during the LGP is believed to have greatly influenced the demographic history of contemporary marine species worldwide (Hewitt, 2004). Considering the topography of the YS, which is characterized by a flat, shallow, and extensive continental shelf (with an average depth of 55 m), population size changes resulting from the LGP are assumed to have been very significant for marine species in this region (reviewed in Ni et al., 2014 and references therein). The China group showed a significant population expansion more recently (after ∼25 kya), likely reflecting the impact of the Last Glacial Maximum about 19–21 kya (Yokoyama et al., 2000). Different population demography, however, was observed for the Japan group, which showed a gradual increase since 550 kya, which is inconsistent with all other groups. We consider this result may be an artifact from the small number of individuals (3–7 individuals/population) included in the analysis, or else this population has its own historical demography. Note that due to the absence of a reliable molecular clock for mitochondrial genes of *A.* cf. *rubrolineata*, the estimated population expansion times of different groups in this study remain speculative. In addition, the expansion times could be more recent if a molecular clock several times faster was applied under the hypothesis of time-dependent molecular rate (Ho et al., 2011). Population genetic analysis of this study is based on a single locus of mtDNA sequence data, and therefore further analysis using multi-locus data from more extensive sampling of the species (particularly additional sampling from Japanese populations) would be required to resolve this uncertainty.

## Conclusion

Chitons represent one of the ancient groups of marine mollusks but have so far attracted little research effort in marine phylogeography (but see Veale & Lavery, 2011). In this study, we investigated genetic diversity and population genetic structure based on two mitochondrial gene fragments (COI and 16S) for *A.* cf. *rubrolineata* populations in the NWP. In contrast to the lack of phylogeographic pattern commonly revealed for other coastal species in the YS, we observed significant population structure (limited gene flow among the regional groups) in *A.* cf. *rubrolineata,* which may be attributed to both life-history features (a short planktonic larval duration) and the warm current system. Nevertheless, within each of our three regions, we also observed genetic homogeneity, which might be maintained by local surface currents and habitat continuity. The phylogeographic pattern revealed in *A.* cf. *rubrolineata* in the present study underscores the interplay of historical and contemporary oceanography (both geography and hydrology) and life-history features on historical demography and population structure in the NWP. Further population genetic studies using multi-locus genetic data are needed to assess the relative importance of biotic and abiotic factors that could more generally influence the phylogeographic patterns of marine species in the YS.

##  Supplemental Information and Declarations

10.7717/peerj.8794/supp-1 Figure S1 Mismatch distribution analyses for the entire population and each regional group The observed and expected mismatch distributions are indicated by a dashed blue line and solid red line, respectively.

## Additional Information and Declarations

### DNA Deposition

The following information was supplied regarding the deposition of DNA sequences: Sequences are accessible via GenBank accession numbers MN205571 to MN205930. However, these are not publicly available yet. Sequences are available for review in the Supplementary Files. This includes the 16S and CO1 sequences.

### Data Availability

The following information was supplied regarding data availability: Data is available in the Supplementary Files and the sequences are available at GenBank: MN205571–MN205930.
