# Supplementary figures and images for: Life-history features and oceanography drive phylogeographic patterns of the chiton Acanthochitona cf. rubrolineata (Lischke, 1873) in the northwestern Pacific
Source: PeerJ. 2020 Apr 8;8:e8794. doi: 10.7717/peerj.8794 (PMC7359822; doi:10.7717/peerj.8794)

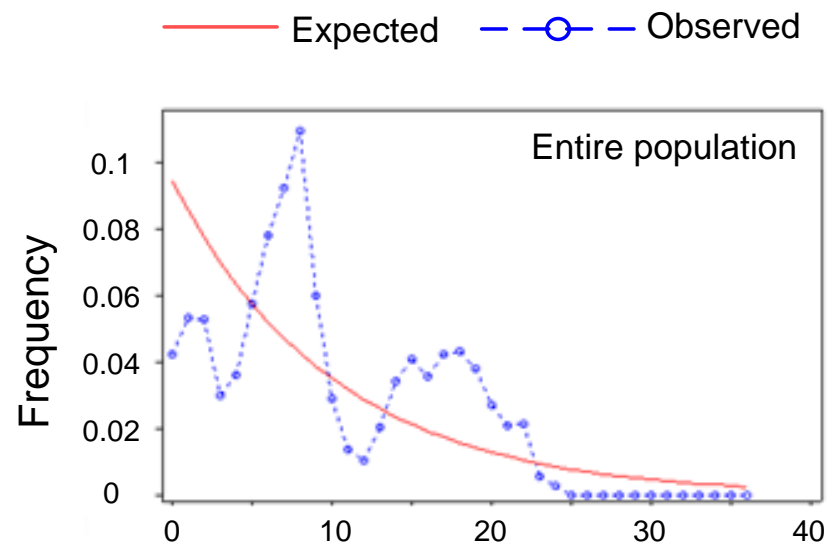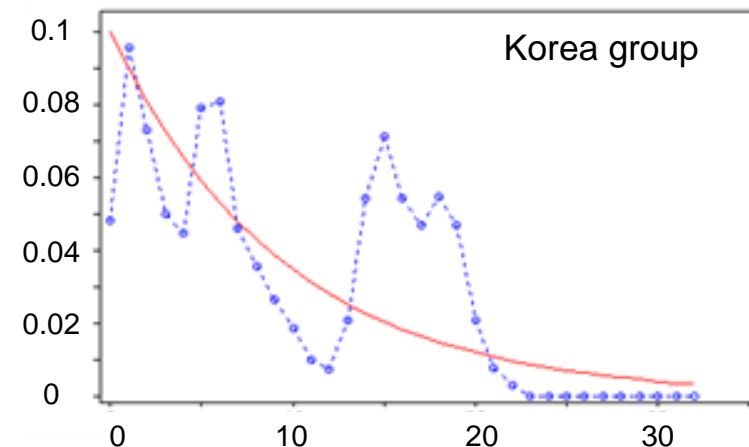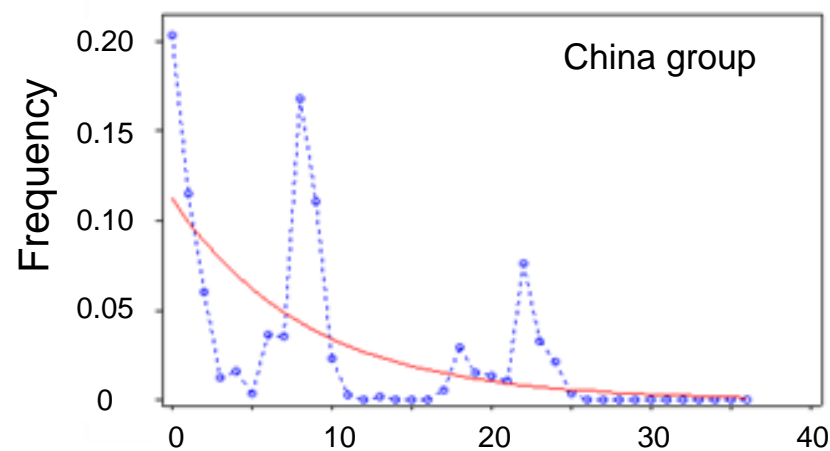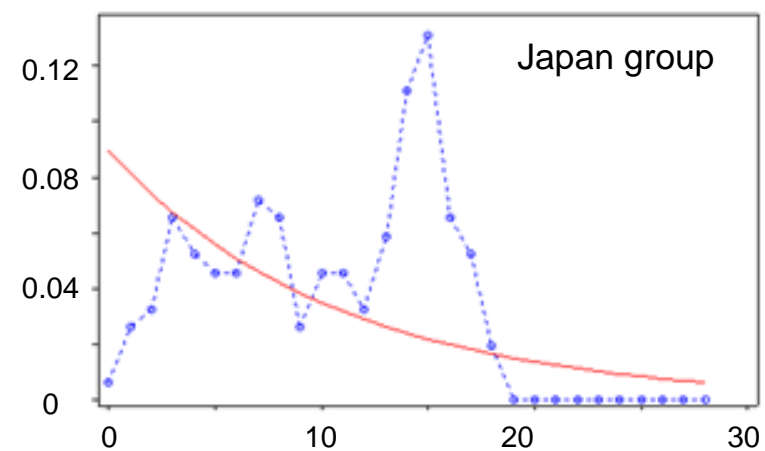

Pairwise differences

Pairwise differences

Supplement: Figure S1 — The observed and expected mismatch distributions are indicated by a dashed blue line and solid red line, respectively. [file peerj-08-8794-s001.pdf]
